# Real-world insights into moderately hypofractionated thoracic radiotherapy in elderly and multimorbid patients with stage II/III NSCLC: a retrospective study

**DOI:** 10.2340/1651-226X.2025.43496

**Published:** 2025-07-25

**Authors:** Helene Kravutske, Sina Mansoorian, Lukas Käsmann, Janina Lehmann, Cedric Richlitzki, Diego Kauffmann-Guerrero, Nina-Sophie Schmidt-Hegemann, Niels Reinmuth, Amanda Tufman, Julien Dinkel, Richard Gaus, Farkhad Manapov, Claus Belka, Chukwuka Eze

**Affiliations:** a Department of Radiation Oncology, LMU University Hospital, LMU Munich, Munich, Germany; b German Cancer Consortium (DKTK), partner site Munich; and German Cancer Research Center (DKFZ), Heidelberg, Germany; c Comprehensive Pneumology Center Munich (CPC-M), Member of the German Center for Lung Research (DZL), Munich, Germany; d Department of Medicine V, University Hospital, LMU Munich, Munich, Germany; e Department of Oncology, Asklepios Lung Clinic Munich-Gauting, Gauting, Germany; f Department of Radiology, University Hospital, LMU Munich, Munich, Germany; g Department of Radiology, Asklepios Lung Clinic Munich-Gauting, Gauting, Germany; h TUM School of Computation, Information and Technology, Munich, Germany; i Bavarian Cancer Research Center (BZKF), Munich, Germany

**Keywords:** Hypofractionated radiotherapy, non-small cell lung cancer, stage II/III, inoperable, frailty

## Abstract

**Purpose:**

Investigating real-world outcomes of moderately hypofractionated radiotherapy (hypoRT) in elderly and multimorbid stage IIB–IIIC non-small-cell lung cancer (NSCLC) patients ineligible for concurrent chemoradiation.

**Methods:**

We retrospectively analysed 70 patients with primary or recurrent stage IIB–IIIC NSCLC (TNM, 8th edition). HypoRT was administered to a total dose of 38–56 Gy in 10–17 fractions (2.5–3.8 Gy/fraction). Patterns of recurrence, survival outcome, and toxicity were assessed.

**Results:**

Seventy patients, with a median age of 76.4 years (range: 51.6–88.2 years), who received hypoRT between August 2015 and September 2022, were reviewed. At baseline, the median Charlson Comorbidity Index (CCI) with oncological diagnosis was 8 (range: 3–13). With a median follow-up post-radiotherapy of 63.9 months (95% Confidence Interval [CI]: 34.8–93.1 months), median progression-free survival (PFS) was 7.6 months (95% CI 6.0–11.0 months), and the median overall survival (OS) was 20.7 months (95% CI 16.7–30.7 months). Competing risk analysis revealed 12-month cumulative incidences of locoregional and distant failure in 41% (95% CI 30–53%) and 14% (95% CI 6–23%) of patients, respectively. Following disease progression, 45 patients received subsequent therapy: 25 underwent additional radiotherapy, 22 received systemic treatment (including immunotherapy), and 19 were referred for best supportive care. Treatment was well tolerated; only 3 patients (4%) developed grade 3 pneumonitis. No adverse events of grade > 3 were reported.

**Interpretation:**

Moderately hypoRT is a safe, feasible, and effective treatment option for elderly and multimorbid patients with stage IIB–IIIC NSCLC, offering encouraging survival outcomes and low toxicity rates. Future prospective studies are needed to validate these findings and optimise treatment strategies for this high-risk population.

## Introduction

Lung cancer is the leading cause of cancer-related deaths worldwide [1]. Upon initial diagnosis, the prognosis of locally advanced (LA) or metastatic non-small-cell lung cancer (NSCLC) is generally poor [2]. The management of LA-NSCLC encompasses a spectrum of therapeutic interventions, including resection, radiation therapy, systemic treatment, their combination, or best supportive care (BSC) [3]. The standard of care for LA-NSCLC generally entails concurrent chemotherapy and conventionally fractionated radiotherapy (CFRT) [4]. The integration of immunotherapy with programmed cell death ligand 1 (PD-L1) inhibitor consolidation by administering durvalumab post chemoradiotherapy has significantly improved progression-free survival (PFS) and overall survival (OS) [5]. However, this treatment approach may pose challenges for multimorbid and elderly patients, particularly those burdened with multiple comorbidities, compromised performance status, and frailty, which are associated with increased toxicity [3]. Consequently, many elderly or multimorbid patients undergo palliative radiotherapy or receive BSC [6].

With the ageing demographic, the importance of tailored therapy for elderly patients assumes increasing significance. Advanced age often coincides with a myriad of comorbidities, influencing treatment tolerance and prognosis in this patient cohort [7–9]. Accelerated hypofractionated radiotherapy (AHRT) is an alternative approach to standard radiation therapy (STRT), aiming to condense overall treatment time (OTT) by administering higher daily biologically effective doses (BEDs). This strategy effectively counters the accelerated repopulation of tumour cells while fostering enhanced patient compliance [10]. Past studies on AHRT primarily enrolled patients with favourable risk profiles [11–13]. However, recent studies have expanded to include those with unfavourable risk factors, poor performance status, and compromised lung function [14–20].

The existing data on treatment for elderly patients with LA-NSCLC is limited, highlighting the need for further research and development in this context. This real-world study sought to investigate the clinical outcomes of moderately hypofractionated radiotherapy (hypoRT) in this multimorbid and elderly patient cohort.

## Materials and methods

Following approval from the institutional review board of the Ludwig Maximilian University of Munich (reference number: 17-230), we retrospectively analysed 70 consecutive patients with histologically or cytologically confirmed NSCLC who underwent moderately hypoRT for primary or recurrent stage IIB–IIIC NSCLC (as per the 8th edition Tumour, Node, Metastasis (TNM) classification) at our institution between August 2015 and September 2022. All cases were discussed at the multidisciplinary tumour board and deemed ineligible for surgery or concurrent chemoradiation due to advanced age, comorbidities, or frailty. The medical records of each patient were reviewed and analysed.

For the initial workup, patients underwent positron emission tomography (PET) with 2-deoxy-(fluorine-18) fluoro-D-glucose integrated with a computer tomography (CT) scan or a CT scan of the chest/upper abdomen. In addition, each patient underwent either a magnetic resonance imaging (MRI) or a contrast-enhanced CT scan of the brain, and baseline pulmonary function parameters were assessed. Based on physicians’ notes, Eastern Cooperative Oncology Group (ECOG) performance status was ascertained, and the Charlson Comorbidity Index (CCI) was calculated. Tumour size and staging were determined through a comprehensive assessment of imaging reports and reanalysis of imaging. The staging was complemented by endobronchial ultrasound (EBUS) guided bronchoscopy for lymph node staging when feasible. Smoking habits were stratified into categories based on pack-years (PY). Furthermore, the baseline assessment encompassed the evaluation of chronic obstructive pulmonary disease (COPD) or other pulmonary diseases.

The gross tumour volume (GTV) encompassed the primary tumour and regionally involved nodes on CT (> 1 cm on short axis) or PET scan (maximum standardised uptake value > 3) or were positive on EBUS. In cases with four-dimensional CT for planning, the internal target volume (ITV) was defined as the GTV and any ventilatory motion. Clinical target volumes (CTVs) extended 0.5 cm beyond the GTV/ITV. The planning target volume (PTV) was 0.5–1 cm beyond the CTV, with the exact margin size determined by the use of four-dimensional CT for planning.

The dose was prescribed so that at least 95% of the GTV/PTV received 95% of the prescription dose. Primarily, intensity-modulated radiation therapy (IMRT) or volumetric modulated arc therapy (VMAT), and in some cases, three-dimensional conformal radiation therapy (3D-CRT), were utilised. Patients received 10–17 fractions with daily doses ranging from 2.5 to 3.8 Gy, totalling 38–56 Gy (BED₁₀: 50.7–75.6 Gy). The most common regimens were 3.0 Gy × 15 (*n* = 22, 31.4%) and 3.0 Gy × 16 (*n* = 32, 45.7%). Less frequent schedules included variations such as 2.5 Gy × 17, 3.5 Gy × 15–16, and 3.8 Gy × 10, each used in a small number of patients (Supplementary Table 1).

Generally, at our institution, acute non-haematological toxicities were evaluated using the National Cancer Institute Common Terminology Criteria For Adverse Events (NCI-CTCAE) version 4.0 before the initiation of radiotherapy, weekly during treatment, and up to 3 months post-treatment (with extended monitoring up to 6 months for pneumonitis). Pulmonary function tests (PFTs) were performed at baseline and, if clinically indicated, after hypoRT. Furthermore, according to national guidelines, CT scans, or whole-body PET/CT scans were conducted every 3 months up to 2 years, biannually during the following 2 years, and annually thereafter.

Tumour progression was assessed via the Response Evaluation Criteria in Solid Tumors (RECIST) version 1.1. Local progression was defined as tumour progression within the high-dose regions of the treated field. Recurrences occurring at the periphery of the treatment field, in the mediastinal/hilar or supraclavicular lymph nodes, or within the ipsilateral lung were categorised as locoregional recurrences. Malignant effusions (pleural and/or pericardial) and metastases, including in the contralateral lung, were classified as distant failures [21]. Two board-certified radiation oncologists carefully reviewed the radiological findings to ascertain the pattern of progression.

The reverse Kaplan-Meier method was used to determine the median follow-up, defined as the time from the end of hypoRT to the last follow-up. Survival data were estimated using the Kaplan-Meier method. Progression-free and overall survival were calculated from the last date of hypoRT to locoregional/systemic progression or death for PFS and death from any cause or last follow-up for OS. Locoregional PFS (LR-PFS) and distant metastasis-free survival (DMFS) were defined as the time to locoregional recurrence/progression and time to distant progression, respectively. A competing risk analysis was performed for locoregional and distant failure, with death as a competing risk event from the end of hypoRT. Toxicity and subsequent therapies were also assessed. Additional patient information was extracted from the medical records, including baseline performance status, smoking status, and lung function parameters.

In this study, we determined the BED to provide a consistent measure of the effect of radiation across different fractionation schedules. Biologically effective dose was calculated using the formula:

BED = n × d (1 + d/α/β), where *n* = the total number of treatment fractions and *d* = the dose administered per fraction. The term α/β is a parameter that reflects the sensitivity of the targeted tissue to radiation and is central to understanding the biological impact of varying radiation doses.

For this study, we used an α/β value of 10 Gy, specific to tumour tissue. By incorporating the total dose and the fraction size into a single metric, BED enables comparisons of therapeutic efficacy across various fractionation schemes, especially when comparing survival, local control, and PFS across different fractionation schemes.

Statistical analyses were performed using IBM SPSS Version 27 (IBM, Armonk, New York) and R version 4.4.0 (R Foundation for Statistical Computing, Vienna, Austria). A significance level of *p* < 0.05 was used to indicate statistically significant differences between the tested groups. Variables such as age, sex, smoking status, CCI, ECOG, histology, and TNM stage were analysed using univariable analysis (UVA) and the log-rank test to identify significant predictors of treatment outcomes. Variables with significant (*p* < 0.05) or borderline significant *p*-values (*p* < 0.1) were further analysed using multivariable Cox regression to determine predictors of OS, PFS, LR-PFS, and DMFS.

## Results

The medical charts of 70 eligible patients treated with hypoRT at our department between August 2015 and September 2022 were reviewed. In this cohort, the median age was 76.4 years (range: 51.6–88.2 years). Of these patients, 24 were aged 80 years or older, 41 were aged 75 years or older, and 54 were aged 70 years or older. In 19 (27.1%) patients, adenocarcinoma was confirmed, and squamous cell carcinoma was confirmed in 43 (61.4%) patients. Four of the remaining eight patients had histology that was not otherwise specified (NOS); two patients were diagnosed with sarcomatoid carcinoma, one patient was diagnosed with an unspecified large cell carcinoma, and one patient had a large cell neuroendocrine carcinoma. A total of 13 patients underwent induction chemotherapy. Two patients received concurrent chemotherapy, while two others received immunotherapy before radiotherapy. In addition, one patient received radiotherapy between immunotherapy cycles. Following hypoRT, seven patients received immunotherapy, including four who received durvalumab, continuing until disease progression or unacceptable toxicity. Of the 70 patients, 65 received IMRT/VMAT; the remaining 5 were treated using 3D-CRT.

At baseline, the median CCI, including the oncologic diagnosis was 8 (range: 3–13). The median value of the mean lung dose (MLD) was 9.6 Gy (range: 1.0–14.2 Gy), with the median V18 for both lungs being 16.8% (range: 1.0–29.5%). The median mean dose to the heart was 5.4 Gy (range: 0.2–14.8 Gy), while the median mean dose to the oesophagus was 15.0 Gy (range: 2.1–26.7 Gy). Patient and treatment characteristics are presented in Table 1.

**Table 1:** Patient and treatment characteristics of the entire cohort.

Characteristic		No.	%
Total		70	100
Age, years	Median (range)	76.4 (51.6–88.2)	
	Mean (SD)	75.6 (7.9)	
	≥ 80	24	34.3
	≥ 75	41	58.6
	≥ 70	54	77.1
	< 70	16	22.9
Sex	Male	54	80.0
	Female	16	20.0
T category	Tx	6	8.6
	T1	9	12.9
	T2	10	14.3
	T3	20	28.6
	T4	25	35.7
N category	N0	17	24.3
	N1	5	7.1
	N2	26	37.1
	N3	22	31.4
Stage	IIB	8	11.4
	IIIA	20	28.6
	IIIB	18	25.7
	IIIC	11	15.7
	Recurrent	13	18.6
Baseline CCI	Median	8 (3–13)	
	Mean (SD)	7.9 (2.1)	
	1–3	1	1.4
	4–6	17	24.3
	≥ 7	52	74.3
Baseline ECOG	Median	1 (0–3)	
	Mean (SD)	1.2 (0.7)	
	0	6	8.6
	1	44	62.9
	2	17	24.3
	3	3	4.3
Staging PET-CT	Yes	62	88.6
	No	8	11.4
Histology	SCC	43	61.4
	ACC	19	27.1
	Other (NOS, sarcomatoid, large cell)	8	11.4
Tumour and treatment volume	GTV Volume, median (IQR)	80.8 cc (29.9–146.8)	
	PTV Volume, median (IQR)	202.0 cc (109.2–406.1)	
Induction treatment	Yes	13	18.6
	No	57	81.4
Subsequent systemic therapy	Yes	22	31.4
	No	48	68.6
Radiation parameters	Median MLD	9.6 Gy (range: 1.0–14.2 Gy)	
	Median V18	16.8% (range: 1.0–29.5%)	
	Median mean heart dose	5.4 Gy (range: 0.2–14.8 Gy)	
	Median mean esophagus dose	15.0 Gy (range: 2.1–26.7 Gy)	

no: number; CCI: Charlson Comorbidity Index with oncological diagnosis; ECOG: Eastern Cooperative Oncology Group; PET-CT: Positron Emission Tomography–Computed Tomography; SD: Standard Deviation; SCC: Squamous Cell Carcinoma; ACC: Adenocarcinoma; MLD: Mean Lung Dose; hypoRT: Hypofractionated Thoracic Radiotherapy; GTV: gross tumour volume; PTV: planning target volume; IQR: Interquartile Range; NOS: not otherwise specified.

**Figure 1:**
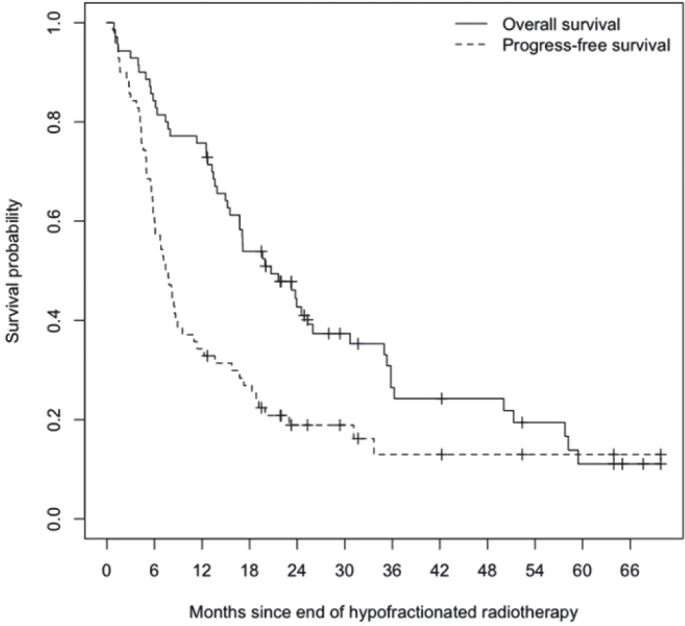
Kaplan-Meier estimate of overall survival (OS) and progression-free survival (PFS). Median OS was 20.7 months (95% CI 16.7–30.7 months). Median PFS was 7.6 months (95% CI 6.0–11.0 months).

**Figure 2:**
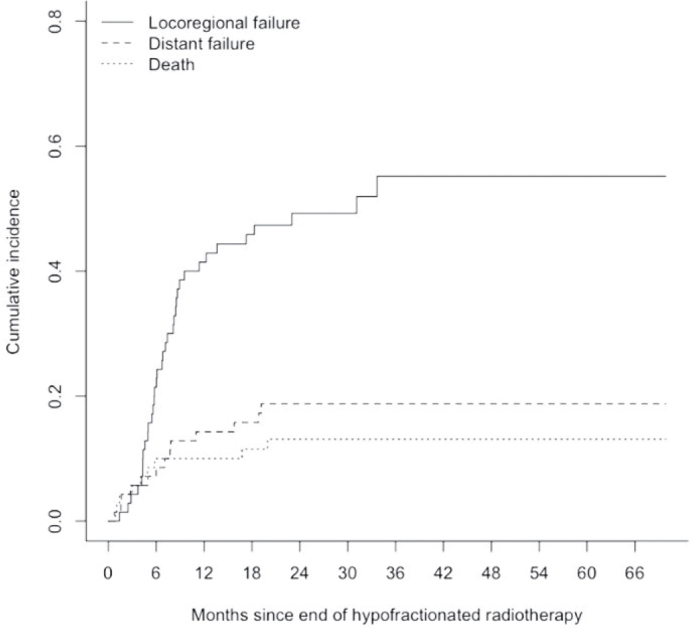
Cumulative Incidence of locoregional failure and distant failure with death as a competing risk event. Twelve-month rate of locoregional failure 41% (95% CI 30–53%) and distant failure 14% (95% CI 6–23%).

With a median follow-up of 63.9 months post-radiotherapy (95% Confidence Interval [CI]: 34.8–93.1 months), the median PFS was 7.6 months (95% CI 6.0–11.0 months), and the median OS was 20.7 months (95% CI 16.7–30.7 months) (Figure 1). The 6- and 12- months PFS rates were 60% (95% CI 50–73%) and 34% (95% CI 25–47%), respectively, while the corresponding OS rates were 84% (95% CI 76–93%) and 76% (95% CI 66–86%), respectively. As of the cutoff date in October 2023, 17 (24%) patients were still alive. Locoregional progression was observed in 40 (57%) patients, while distant progression occurred in 27 (39%) patients. With death considered as a competing risk event, locoregional and distant failure rates were 41% (95% CI 30–53%) and 14% (95% CI 6–23%), respectively after 12 months (Figure 2).

In the UVA, ECOG-PS was a statistically significant prognostic factor for OS (*p* = 0.005) and PFS (*p* = 0.014). In addition, histology was a significant predictor of PFS (*p* = 0.022) and LR-PFS (*p* = 0.004). For DMFS, significant predictors included smoking history (*p* = 0.045) and BED_10_ (*p* = 0.042). Tumour stage (*p* = 0.06) and CCI (*p* = 0.052) were borderline predictors of OS, and BED_10_ was a borderline predictor of PFS (*p* = 0.071). Patients with GTV below the median demonstrated significantly better outcomes in UVA, including OS (*p* = 0.012) and PFS (*p* = 0.044). A trend towards improved LR-PFS (*p* = 0.09) and DMFS (*p* = 0.06) was also observed. In MVA, higher ECOG-PS was associated with worse OS (Hazard Ratio (HR) 2.218, 95% CI: 1.220–4.033; *p* = 0.009) and PFS (HR 1.930, 95% CI: 1.046–3.559; *p* = 0.035). CCI ≥10 was predictive of inferior OS (HR 3.125, 95% CI: 1.316–7.419; *p* = 0.010). GTV ≥ median correlated with shorter PFS (HR 2.061, 95% CI: 1.16–3.67; *p* = 0.014). Adenocarcinoma (ACC) histology was associated with improved LR-PFS versus SCC (HR 0.296, 95% CI: 0.12–0.72; *p* = 0.008). A detailed summary of univariable and multivariable analyses is presented in Tables 2 and 3.

**Table 2:** Univariable analysis of the entire cohort regarding OS, PFS, LR-PFS, and DMFS.

Variable	Category	No. of Patients	Univariable analysis: *p*-value			
			OS	PFS	LR-PFS	DMFS
Age, years			0.472	0.448	0.715	0.468
	< 70 70–74 75–79 ≥ 80	16 13 17 24				
Sex			0.947	0.531	0.939	0.537
	Male Female	54 16				
Stage			***0.060***	0.363	0.792	0.104
	IIB–IIIA IIIB–IIIC	41 29				
T category			0.124	0.219	0.127	0.690
	TX-T2 T3–T4	25 45				
N category			0.779	0.748	0.900	0.322
	N0/N1 N2 N3	22 26 22				
Smoking history, py			0.488	0.759	0.347	0.045*
	0–30 30–75 ≥ 75	25 35 10				
ECOG-PS			0.005*	0.014*	0.513	0.403
	0–1 2–3	50 20				
CCI			***0.052***	0.471	0.646	0.409
	1–6 7–9 ≥ 10	18 37 15				
Histology			0.241	0.022*	0.004*	0.107
	SCC ACC Other	43 19 8				
IC			0.772	0.794	0.236	0.298
	Yes No	13 57				
BED_10_			0.238	***0.071***	0.214	0.042*
	< 62.4 Gy ≥ 62.4 Gy	28 42				
GTV Volume	< median ≥ median	35 35	0.012*	0.044*	**0.094**	**0.060**

* *p* < 0.05

CCI: Charlson Comorbidity Index; PFS: progression-free survival; OS: overall survival; BED_10_: biologically effective dose (assuming α/β = 10); ECOG: Eastern Cooperative Oncology Group; GTV: gross tumour volume; LR-PFS: Locoregional PFS; DMFS: distant metastasis-free survival.

The bold italic values indicate borderline statistically significant findings (p < 0.1) in the univariable analysis

**Table 3:** Multivariable analysis of the entire cohort regarding OS, PFS, LR-PFS, and DMFS.

Variable	Category	No. of Patients	Multivariable Analysis: HR ([95% CI], *p*-value)			
			OS	PFS	LR-PFS	DMFS
**Stage**						
	IIB–IIIA	41	1.0 (ref)			
	IIIB–IIIC	29	***HR 1.610 (95% CI: [0.862–3.006], p = 0.135)***			
**Smoking history, py**						
	0–30	25				1.0 (ref)
	30–75	35				HR 0.813 ([0.377–2.151], *p* = 0.813)
	≥ 75	10				HR 0.900 ([0.377–3 2.151], *p* = 0.076)
**ECOG-PS**						
	0–1	50	1.0 (ref)	1.0 (ref)		
	2–3	20	HR 2.218 (95% CI: [1.220–4.033], p = 0.009*)	HR 1.930 ([1.046–3.559], *p* = 0.035*)		
**CCI**						
	1–6	18	1.0 (ref)			
	7–9	37	HR 1.364 (95% CI: [0.676–2.750], *p* = 0.386)			
	≥ 10	15	HR 3.125 (95% CI: [1.316–7.419], p = 0.010*)			
**Histology**						
	SCC	43		1.0 (ref)	1.0 (ref)	
	ACC	19		HR 0.845 ([0.433–1.650], *p* = 0.622)	HR 0.296 ([0.121–0.723], *p* = 0.008*)	
	Other	8		HR 1.568 ([0.622–3.953], *p* = 0.341)	HR 1.762 ([0.608–5.106], *p* = 0.297)	
**BED**_10_						
	< 62.4 Gy	28		1.0 (ref)		1.0 (ref)
	≥ 62.4 Gy	42		HR 0.697 ([0.402-1.210, *p* = 0.199])		HR 1.272 ([0.530–3.055], *p* = 0.591)
**GTV**						
	< median	35	1.0 (ref)	1.0 (ref)	1.0 (ref)	1.0 (ref)
	≥ median	35	**HR 1.706 (95% CI: [0.914–3.183], *p* = 0.093)**	HR 2.061 (95% CI: [1.158–3.667], *p* = 0.014*)	HR 1.643 (95% CI: [0.847–3.185], *p* = 0.142)	HR 1.434 (95% CI: [0.633–3.245], *p* = 0.388)

* *p* < 0.05

no.: number; OS: overall survival; PFS: progression-free survival; LR-PFS: locoregional progression-free survival; DMFS: distant metastasis-free survival; PY: pack years; ECOG: Eastern Cooperative Oncology Group; CCI: Charlson comorbidity index; SCC: squamous cell carcinoma; ACC: adenocarcinoma; IC: Induction Chemotherapy; BED_10_: biologically effective dose (assuming α/β = 10); GTV: gross tumour volume.

The bold italic values indicate statistically significant findings (p < 0.05) in the multivariable analysis

After treatment failure, 45 patients (64%) received subsequent therapy. Among these patients, 25 (56%) underwent at least one additional course of radiotherapy, and 22 (49%) received subsequent systemic therapy, including checkpoint inhibitors or chemotherapy. A second course of irradiation was administered in 11 patients (15.7%) to the in-field thoracic region, in 10 patients (14.3%) in the out-of-field thoracic region, in 4 patients (5.7%) to the brain, and in 1 patient (1.4%) for bone metastases. Best supportive care was recommended for 19 (42%) patients.

Severe toxicities were rare, with only three cases (4%) of grade 3 pneumonitis and no other grade 3 or higher toxicities, as detailed in Table 4.

**Table 4:** Pre-treatment and post-treatment toxicity.

Toxicity grade	Pneumonitis (%)	Oesophagitis (%)	Dermatitis (%)	Dyspnoea		Cough		Dysphagia		Fatigue	
				Baseline (%)	Post-hypoRT (%)	Baseline (%)	Post-hypoRT (%)	Baseline* (%)	Post-hypoRT (%)	Baseline (%)	Post-hypoRT (%)
**I**	46 (65)	4 (6)	4 (6)	24 (34)	27 (38)	30 (42)	41 (58)	2 (3)	19 (27)	6 (8)	15 (21)
**II**	8 (11)	2 (3)	- -	18 (25)	24 (34)	2 (3)	5 (7)	- -	10 (14)	- -	11 (15)
**III**	3 (4)	- -	1 (1)	1 (1)	4 (6)	- -	- -	1 (1)	1 (1)	- -	- -
**IV**	-	-	-	-	-	-	-	-	-	-	-
**V**	-	-	-	-	-	-	-	-	-	-	-

* Three patients reported symptomatic dysphagia before treatment. In two cases, dysphagia was most likely tumour related. One patient suffered from grade 3 dysphagia due to a previous history of oropharyngeal carcinoma.

hypoRT: hypofractionated radiotherapy.

## Discussion

Our study focuses on elderly and multimorbid patients with stage IIB–IIIC NSCLC, often excluded from clinical trials due to frailty or poor performance status. The patient cohort in this study was ineligible for radical chemoradiation, as it primarily consisted of older individuals and those with significant comorbidities or poor performance status. Specifically, 24 patients (34%) were aged ≥ 80 years, while 30 patients (43%) were aged between 70 and 79 years. In addition, 20 patients (29%) had an Eastern ECOG performance status of 2–3, and 52 patients (74%) had a CCI-score of 7 or more.

The potential benefit of chemoradiation for this patient cohort remains controversial. Several studies have shown that while concurrent chemoradiation can enhance OS, it also increases toxicity. This approach is often unsuitable for older patients with poor performance status. Hansen et al. showed that while younger patients experienced advantages from chemoradiation, the additional chemotherapy did not confer survival benefits to those aged 70 years and older [22]. Conversely, in a randomised study, Atagi et al. reported a survival benefit from concurrent chemoradiation in elderly patients [23]. It is essential to acknowledge that in this study, the group receiving chemoradiation comprised predominantly physically fitter elderly patients with better performance status.

In our study, the median PFS was 7.6 months, and the median OS was 20.7 months. The 6- and 12-month PFS rates were 60% and 34%, respectively, while the OS rates were 84% and 76%, respectively. The 12-month locoregional failure at 41% (95% CI 30–53%) and distant failure at 14% (95% CI 6–23%) highlight the importance of achieving effective locoregional control in this high-risk population. Locoregional failure was notably more common than distant failure, suggesting that improving locoregional control – possibly through dose escalation or advanced radiation techniques – may improve outcomes in elderly and multimorbid patients who are not suitable for aggressive systemic therapies [24–26]. The low distant failure rate (14%) may be due to standard national follow-up imaging limited to CT of the chest/upper abdomen in most cases, and may be indicative of hypoRT in combination with contemporary systemic treatments for example immunotherapy acting as a ‘stabiliser’ of systemic disease. A phenomenon that we hypothesised in a previous analysis on patients receiving chemoradiation and consolidation durvalumab [27].

Our survival results are consistent with other studies on elderly patients with LA NSCLC, some of whom received more aggressive treatments, which can be associated with higher toxicity rates [28–30]. The DUART phase II study currently evaluates durvalumab after radiotherapy in 102 patients with stage III NSCLC who were ineligible for chemotherapy, with a median age of 79 years. The study’s final analysis shows a median PFS of 7.6/10.3 months and an OS of 16.8/21.1 months, depending on the radiotherapy dose (40 to < 54 Gy or bioequivalent dose vs. 60 Gy ± 10% or bioequivalent dose). Notably, the rate of grade 3/4 therapy-related adverse events (AEs) within 6 months, which is the study’s primary endpoint, was 9.8% (95% CI: 4.8–17.3%). In comparison, our study demonstrated a lower toxicity profile, with only 4% of patients developing grade 3 pneumonitis, highlighting the favourable tolerability of hypoRT [31].

Similarly, the SPIRAL-RT phase II study investigated radiotherapy followed by durvalumab, reporting a median OS of 20.8 months and PFS of 8.9 months [32]. Despite 81.4% of patients not receiving induction treatment and 68.6% of our patients not receiving subsequent systemic therapy, the observed outcomes (OS: 20.7 months; PFS: 7.6 months) compare favourably with those reported in these studies, albeit retrospective in nature. In our previous analysis of stage IIB–IIIC NSCLC patients treated with hypoRT, we observed median PFS and OS of 10.4 and 18.3 months, respectively, in a high-risk cohort with compromised pulmonary function (median Forced Expiratory Volume In 1 Second [FEV1]: 1.17 L, Diffusion Capacity of the Lungs for Carbon Monoxide [DLCO]-SB: 35%) [18]. These findings align with our current study, both showing favourable toxicity profiles with no grade > 3 AEs. A randomised trial comparing moderate hypofractionation (60 Gy/15 fx) with normofractionation (60 Gy/30 fx) reported a higher mortality rate (10%) in the hypofractionation arm [17]. Cacicedo et al.’s multicentre analysis of elderly LA-NSCLC patients found that only 20% of those aged ≥ 75 years received aggressive treatments like chemoradiation, with most receiving sequential therapies or RT alone. Poor baseline quality of life (QOL), comorbidities, and factors such as pre-existing heart disease and low physical functioning were associated with shorter survival, highlighting the challenges in treating this population [30].

Our study underscores the crucial impact of general health status and comorbidities on outcomes in elderly patients with NSCLC, suggesting these factors may be more influential than traditional prognostic markers typically relevant in younger populations. ECOG-PS and CCI emerged as consistent predictors of survival, offering valuable guidance for individualised treatment decisions and accurate prognostication. In UVA, ECOG-PS was significantly associated with both OS (*p* = 0.005) and PFS (*p* = 0.014), while CCI showed a borderline association with OS (*p* = 0.052). Tumour stage also approached significance for OS (*p* = 0.06). These findings emphasise the combined influence of functional status, comorbidity burden, and disease extent on prognosis.

In multivariable analysis, key clinical variables were significantly associated with survival outcomes. A higher ECOG performance status [2–3] was predictive of worse OS (HR 2.218, 95% CI: 1.220–4.033; *p* = 0.009) and PFS (HR 1.930, 95% CI: 1.046–3.559; *p* = 0.035). Similarly, a high comorbidity burden (CCI ≥10) was associated with poorer OS (HR 3.125, 95% CI: 1.316–7.419; *p* = 0.010), reinforcing the dominant role of patient-related factors in this population. In terms of locoregional control, adenocarcinoma histology demonstrated a favourable impact on LR-PFS compared to squamous cell carcinoma (HR 0.296, 95% CI: 0.121–0.723; *p* = 0.008), in line with previous reports [2, 3]. Tumour volume also emerged as a significant predictor, with GTV ≥ median associated with inferior PFS (HR 2.061, 95% CI: 1.158–3.667; *p* = 0.014), suggesting a role for volumetric parameters in risk stratification. The strong and consistent predictive value of ECOG-PS across multiple endpoints highlights its clinical relevance, regardless of age. When considered alongside CCI, it provides a practical framework for tailoring treatment strategies based on both performance status and comorbidity burden. These findings are consistent with large-scale studies confirming the prognostic significance of ECOG-PS in various cancer treatment settings [5, 6].

The irradiation of LA NSCLC portends lung toxicity, which is particularly concerning for elderly patients. However, dosimetric parameters in this study were acceptable, demonstrating their importance in minimising toxicity in this vulnerable population. Following hypoRT, 11% of patients developed Grade 2 pneumonitis and 4% experienced Grade 3 pneumonitis. Importantly, all cases were either self-limiting or successfully treated, and no instances of pneumonitis greater than Grade 3 were observed, highlighting the overall safety and tolerability of the treatment. No other severe toxicities were reported.

The limitations of the study include a small sample size of 70 patients, reducing the statistical power to detect significant outcomes. The heterogeneity of the patient population, consisting of primary and recurrent NSCLC cases at various stages, complicates interpretation. Being a monocentric study, the results reflect the protocols of a single centre, which limits generalisability. Its retrospective design introduces potential selection bias, as records may not capture all relevant variables. Lastly, the absence of randomisation prevents control for confounding factors, limiting the assessment of true treatment efficacy.

In conclusion, our study underscores the potential of hypoRT to yield favourable outcomes, particularly in multimorbid, frail patients with stage IIB-IIIC NSCLC who cannot undergo more aggressive treatments. With encouraging survival outcomes and low toxicity rates, hypoRT offers a feasible approach for managing this high-risk population. These findings underscore the importance of tailoring treatment to individual patient characteristics, including performance status and comorbidities, and suggest that hypoRT may serve as a foundation for integrating systemic therapies to enhance outcomes. Given the ageing population and increased cancer risk among older patients, it is crucial to include these underrepresented patients in prospective studies and randomised clinical trials.

## Supplementary Material



## Statements & declarations

### Data availability

Due to sensitivity open availability of the supporting data to this study’s findings cannot be granted but are available from the corresponding author upon reasonable request. Data are in controlled access data storage at LMU University Hospital.
