# Supplementary material for: Real-world insights into moderately hypofractionated thoracic radiotherapy in elderly and multimorbid patients with stage II/III NSCLC: a retrospective study
Source: Acta Oncol. 2025 Jul 25;64:43496. doi: 10.2340/1651-226X.2025.43496 (PMC12308537; doi:10.2340/1651-226X.2025.43496)
Supplement: Supplementary file 1 [file AO-64-43496-s1.pdf]

Supplementary material has been published as submitted. It has not been copyedited, or typeset by Acta Oncologica

Supplementary Table 1 Radiation fractionation frequencies

Fractionation (Gy x n)	Total Dose (Gy)	BED_{10} (Gy)	Numbers	Percentage (%)
**2.5 x 17**	42.5	53.1	1	1.4%
**2.8 x 16**	44.8	57.3	1	1.4%
**3.0 x 13**	39.0	50.7	1	1.4%
**3.0 x 14**	42.0	54.6	2	2.9%
**3.0 x 15**	45.0	58.5	22	31.4%
**3.0 x 16**	48.0	62.4	32	45.7%
**3.0 x 17**	51.0	66.3	1	1.4%
**3.3 x 16**	52.8	70.2	2	2.9%
**3.5 x 15**	52.5	70.9	4	5.7%
**3.5 x 16**	56.0	75.6	3	4.3%
**3.8 x 10**	38.0	52.4	1	1.4%
